# Neurophysiological features of STN LFP underlying sleep fragmentation in Parkinson’s disease

**DOI:** 10.1136/jnnp-2023-331979

**Published:** 2024-11-18

**Authors:** Guokun Zhang, Huiling Yu, Yue Chen, Chen Gong, Hongwei Hao, Yi Guo, Shujun Xu, Yuhuan Zhang, Xuemei Yuan, Guoping Yin, Jian-guo Zhang, Huiling Tan, Luming Li

**Affiliations:** 1 National Engineering Research Center of Neuromodulation, Tsinghua University School of Aerospace Engineering, Beijing, China; 2 Peking Union Medical College Hospital, Beijing, China; 3 Department of Neurosurgery, Qilu Hospital of Shandong University Qingdao, Qingdao, Shandong, China; 4 Department of Otolaryngology Head and Neck Surgery, Beijing Tsinghua Changgung Hospital, Beijing, China; 5 Beijing Tiantan Hospital, Beijing, China; 6 Nuffield Department of Clinical Neurosciences, University of Oxford, Oxford, UK; 7 IDG/McGovern Institute for Brain Research at Tsinghua University, Beijing, China

## Abstract

**Background:**

Sleep fragmentation is a persistent problem throughout the course of Parkinson’s disease (PD). However, the related neurophysiological patterns and the underlying mechanisms remained unclear.

**Method:**

We recorded subthalamic nucleus (STN) local field potentials (LFPs) using deep brain stimulation (DBS) with real-time wireless recording capacity from 13 patients with PD undergoing a one-night polysomnography recording, 1 month after DBS surgery before initial programming and when the patients were off-medication. The STN LFP features that characterised different sleep stages, correlated with arousal and sleep fragmentation index, and preceded stage transitions during N2 and REM sleep were analysed.

**Results:**

Both beta and low gamma oscillations in non-rapid eye movement (NREM) sleep increased with the severity of sleep disturbance (arousal index (ArI)-beta_NREM_: r=0.9, p=0.0001, sleep fragmentation index (SFI)-beta_NREM_: r=0.6, p=0.0301; SFI-gamma_NREM_: r=0.6, p=0.0324). We next examined the low-to-high power ratio (LHPR), which was the power ratio of theta oscillations to beta and low gamma oscillations, and found it to be an indicator of sleep fragmentation (ArI-LHPR_NREM_: r=−0.8, p=0.0053; ArI-LHPR_REM_: r=−0.6, p=0.0373; SFI-LHPR_NREM_: r=−0.7, p=0.0204; SFI-LHPR_REM_: r=−0.6, p=0.0428). In addition, long beta bursts (>0.25 s) during NREM stage 2 were found preceding the completion of transition to stages with more cortical activities (towards Wake/N1/REM compared with towards N3 (p<0.01)) and negatively correlated with STN spindles, which were detected in STN LFPs with peak frequency distinguishable from long beta bursts (STN spindle: 11.5 Hz, STN long beta bursts: 23.8 Hz), in occupation during NREM sleep (β=−0.24, p<0.001).

**Conclusion:**

Features of STN LFPs help explain neurophysiological mechanisms underlying sleep fragmentations in PD, which can inform new intervention for sleep dysfunction.

**Trial registration number:**

NCT02937727.

## Introduction

Parkinson’s disease (PD) is one of the most prevalent neurodegenerative diseases predominately affecting dopaminergic system in basal ganglia (BG). Besides prominent movement disorders such as bradykinesia, rigidity, tremor and balance disturbance, more than 80% patients with PD suffer from sleep disturbance.^1 2^ Sleep fragmentations, in particular, correlate with sleep-maintenance insomnia and excessive daytime sleepiness, can further interact with cognitive impairment and accelerated disease progression.^3–6^However, the neurophysiological mechanism of sleep fragmentation in human BG remains largely unknown and results in obstacles towards developing sleep-specific intervention.

A recent study based on non-human primate model of PD suggested that increased beta oscillations (13–30 Hz) in the BG during NREM sleep in Parkinsonian state were associated with sleep fragmentation and reduction in slow wave activities.^7^ However, evidence is still missing in human patients, as most existing studies with local field potential (LFP) recordings from human patients show that the beta oscillation was significantly reduced during NREM sleep compared with REM or awake.^8 9^ Moreover, the wide spread sleep spindles during NREM stage 2 sleep were found in different BG nucleus in healthy non-human primate.^10^ Whether they are dissociable or are interfered by pathological beta bursts, the frequency of which were overlapped with spindles, remained unclear. Thus, gaining more knowledge on this topic will improve our understanding of the mechanism.

Most previous studies with human patients showed how LFPs in BG change with circadian rhythms or sleep stages.^9 11 12^ However, sleep fragmentation was often measured by sleep fragmentation index (SFI) and arousal index (ArI). SFI was calculated as the sum of the number of transitions from sleep to wake and transitions from N3, N2, and REM sleep stages to N1, subsequently divided by the total duration of sleep (measured in hours).^13^ ArI was calculated as the number of short duration arousals per hour of sleep.^13^ Therefore, better understanding of the neurophysiological mechanism of sleep-to-wake and sleep-stage transitions is important to understand sleep fragmentation better.

In our previous studies, we have reported a novel design of a DBS system combining DBS therapy as well as concurrent measurement and real-time LFP transmission together.^14 15^ Here, based on this technology, we conducted simultaneous polysomnography (PSG) monitoring together with subthalamic nucleus (STN) LFP recording during a whole-night sleep in 13 patients with PD to investigate the neurophysiology mechanism of STN LFPs underlying sleep fragmentation in PD.

## Methods

### Participants and clinical evaluation

**Figure 1:**
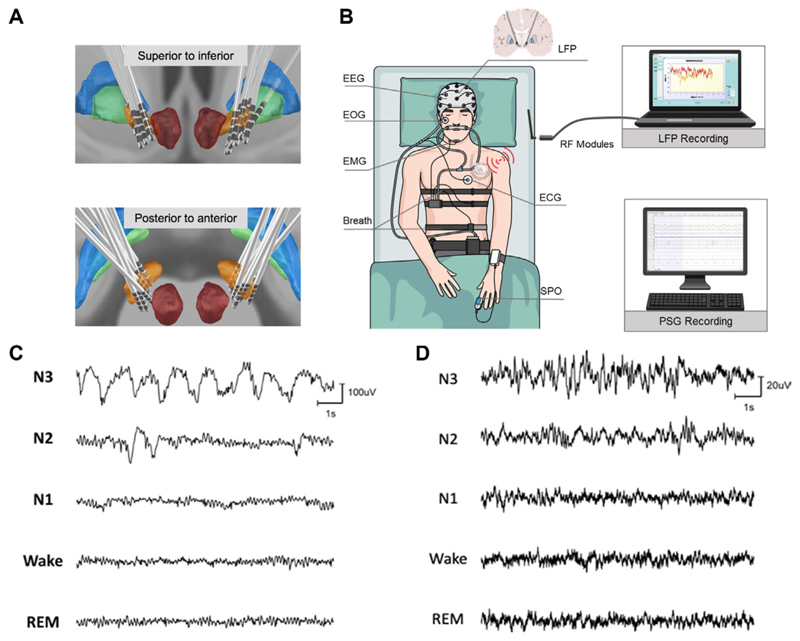
Lead localisation, schematic of recording platform and waveform examples of electroencephalogram (EEG) and subthalamic nucleus (STN) local field potential (LFP). (A) Locations of electrodes from all 12 patients, for whom the LFPs were analysed, were reconstructed by lead DBS and viewed from superior to inferior and posterior to anterior. (B) The polysomnography system consisted of six channels of EEG, submental electromyogram (EMG), electrooculogram (EOG) and ECG recordings. LFPs were recorded through the chronically implanted sensible DBS system and transmitted wirelessly to a PC with radio-frequency (RF) modules. (C, D) Example waveforms of five sleep stages of EEG (C) and STN LFP signal (D) obtained from a single subject.

A total of 13 patients(8 males) with PD with the average age of 56±6.7 years old and average disease duration of 9.5±4.3 years were recruited in this study. All patients had signed informed consent in line with the Declaration of the Principles of Helsinki prior to surgery. All patients were bilaterally implanted in STN with electrodes consisting of four contacts (Model L301C, Beijing PINS Medical Co, China) and a sensing-enabled neurostimulator (G106R, Beijing PINS Medical Co, China). Locations of the electrodes were identified based on presurgical structural MRI and postsurgical CT and reconstructed with Lead DBS tool^16^ as shown in figure 1a.

Clinical evaluation including Unified PD Rating Scale motor score (UPDRS III), Hoehn and Yahr Scale and Minimum Mental State Examination (MMSE) was conducted. One participant (subject 8) was excluded from the STN LFP analysis due to abnormal impedance of the contacts (>100 kΩ), but was included in the analysis of sleep parameters. Demographic characteristics and clinical details and the patients included in the analysis are summarised in table 1.

### Recording

Experiments were conducted 1 month after the DBS surgery before initial programming in a fully equipped sleep lab at the hospital. Patients were in off-DBS and off-medication state. None of the patients involved in this study took drugs for sleep during the period of our research.

Together with STN LFP recording, subjects underwent a whole-night PSG monitoring (figure 1b). Signals of STN LFP were recorded wirelessly using a sensible DBS system and dedicated software (G106R, Beijing PINS Medical Co, China). LFP signals were sampled at 500 Hz after filtered with an RC filter of 0.3–250 Hz implemented on board. The distance of wireless transmission was up to 2 m, avoiding the disturbance of normal sleep caused by LFP recording platform. The LFP recordings were synchronised with the PSG recordings through manually noting the recording starting time of each recording system. Therefore, the resolution of the synchronisation between the two recording systems is about 1 s. More details are listed in online supplemental methods.

### Sleep evaluation

The PSG recording was evaluated by two sleep experts, who independently labelled each 30s-epoch data as Wake, REM sleep, N1, N2 or N3 according to the manual guideline from the American Academy of Sleep Medicine. Only the epochs that had the same labelling from the two experts were considered in this study. Measurement of sleep fragmentation and analysis of EMG and EOG were listed in online supplemental methods.

### Data processing

For EEG signals, C3-M2 and C4-M1 channels were selected for analysis after resampled at 256 Hz. Considering the volume conduction minimisation and signal references for closed-loop DBS, we constructed a bipolar LFP signal for each hemisphere based on recordings from pairs of contacts adjacent to the contact used for therapeutic stimulation. In cases where the stimulation contact was at the end of the electrode (either contact 0 or 3, see table 1), the bipolar channel that showed the highest beta peak in the power spectrum density (PSD) when the patients were at rest and awake was used for further analyses.

EEG and LFP signals were preprocessed to reduce movement artefacts and 50 Hz power line interference (see online supplemental methods). Moving window short-time Fourier transform was used to estimate PSD of the EEG and LFP signals of each time point, with 2 s hamming window, 95% overlap and a frequency resolution of 1 Hz. Average power of typical oscillation bands (delta, 1–3 Hz; theta, 4–7 Hz; alpha 8–14 Hz; beta, 15–30 Hz; low gamma, 31–60 Hz) was then extracted.^17^

### Spindle and beta burst determination

In order to investigate the relationship between the beta bursts and sleep spindles during NREM sleep, in this study, both spindle and beta bursts detections were only performed on hemispheres with prominent beta (10–35 Hz) or spindle activity (10–17 Hz) during Wake and NREM, specifically, identified using FOOOF (Fitting Oscillations and One-Over-F) algorithm^18^ (more details in the online supplemental material). Periodic oscillatory activities in spindle and beta frequency bands were identified in 11 and 18 out of the 24 recorded hemispheres, respectively. This resulted in 11 out of the 24 hemispheres (from nine patients) showed both distinct peak in the spindle frequency band during NREM sleep and the beta frequency band during awake.

Spindle events detection was performed with an algorithm which was widely applied and verified in previous studies of LFP and EEG.^10 19^ Beta bursts were identified as previously illustrated.^20^ Only beta bursts longer than 250 ms were included in our analysis. See more details in online supplemental methods.

### Statistical analysis

We employed the Wilcoxon rank-sum test to compare the electrophysiological activities between different sleep stages. FDR (false discovery rate) correction was performed in multiple comparisons. Correlation analysis in this study was conducted by Spearman’s rank correlation coefficient. Linear mixed-effect models (LMM) were used to compare the differences in electrophysiological characteristics. In each comparison, recorded hemispheres were considered as random effects to compensate multiple measurements within and between hemispheres. All data processing and statistical analysis were performed in MATLAB 2021b.

## Results

### Abnormal sleep architectures correlated with clinical impairment in patients with PD

**Figure 2:**
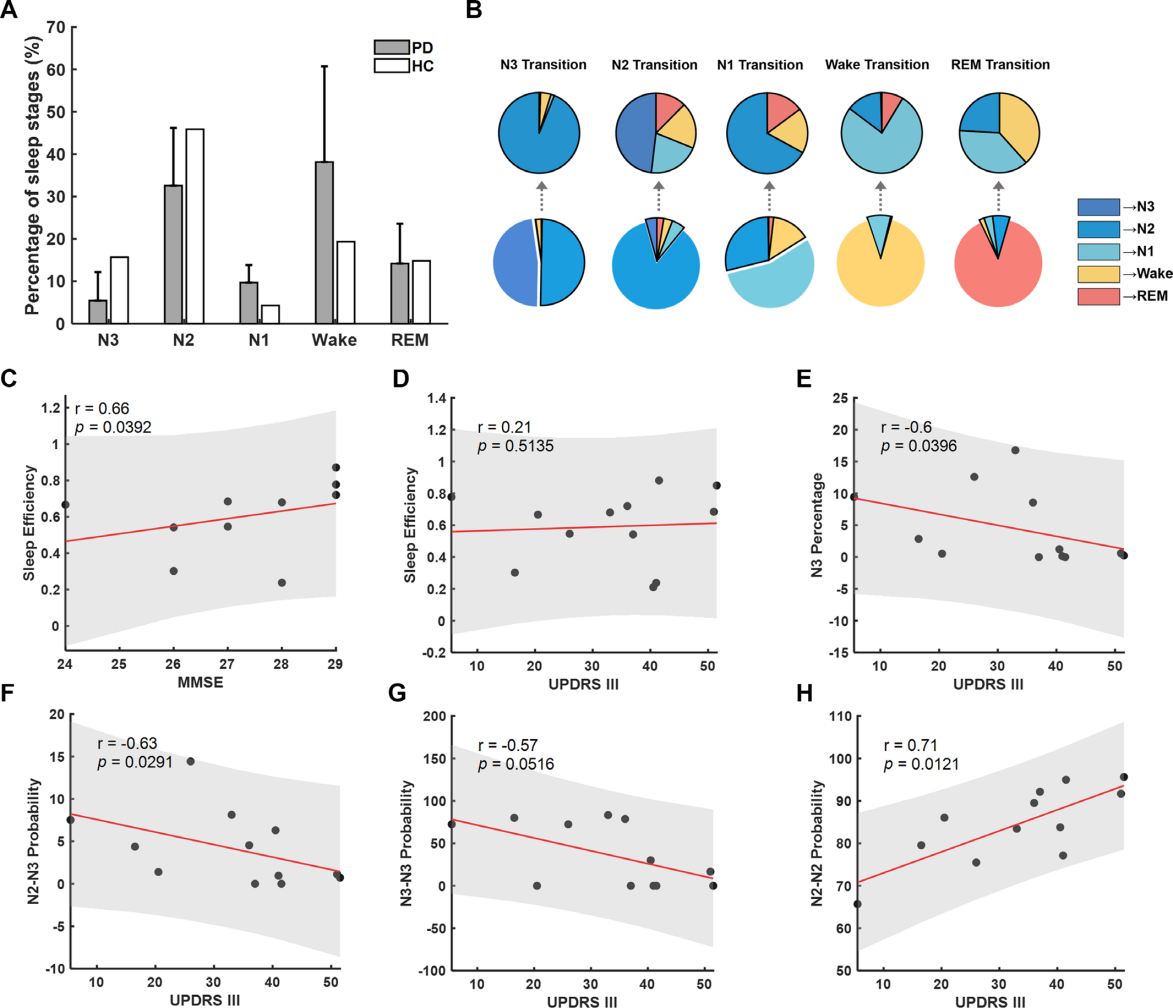
Sleep parameters and clinical correlation. (A) Comparison of sleep stage percentage between Parkinson patients (grey bars) and health control (white bars) within the similar age group. The grey bar illustrated the mean±SD of sleep stage percentage from 13 patients. (B) Average sleep stage transition probabilities across sleep stages. The pie charts in the first row presented the probabilities of sleep transition from one sleep stage to another different sleep stages; the second raw presented the probabilities of each sleep stage transitioning to another sleep stage in the following epoch. (C) Correlations between sleep efficiency and Minimum Mental State Examination (MMSE). (D) Correlations between sleep efficiency and Unified PD Rating Scale motor score (UPDRS III). (E) Correlations between N3 percentage and UPDRS III. (F) Correlations between N2–N3 transition probability and UPDRS III. (G) Correlations between N3–N3 transition probability and UPDRS III. (H) Correlations between N2–N2 transition probability and UPDRS III. In (C–H), each dot represents data from one participant; the red solid line and grey shading indicate the linear fitting and 95% CIs.

The percentage of different sleep stage times during the whole-night sleep was analysed according to PSG evaluation. Compared with health control within the similar age group as shown in previous research,^21^ patients with PD showed a tendency of increased percentage in Wake and NREM Stage 1 (N1) and decreased percentage in NREM Stage 3 (N3). Across recorded participants, the sleep efficiency of this recorded night correlated with cognitive function, as measured by the score of Minimum Mental State Examination (MMSE) tested the day before PSG monitoring (n=10, r=0.7, p=0.0392, figure 2c), but not with UPDRS III (n=12, r=0.2, p>0.1, figure 2d). Correlation analysis showed that N3 percentage and N3 transition patterns, including the N2-to-N3 transition probability and N3 stability, all negatively correlated with UPDRS III score (n=12, r=−0.6, p=0.0396, figure 2e for N3 percentage; r=−0.6, p=0.0291, figure 2f for N2-to-N3 transition probability; and r=−0.6, p=0.0516, figure 2g for N3 stability), indicating the essential association between motor impairment and shortened and fragmented N3 sleep.

### Sleep-stage-dependent characteristics of STN LFP

**Figure 3:**
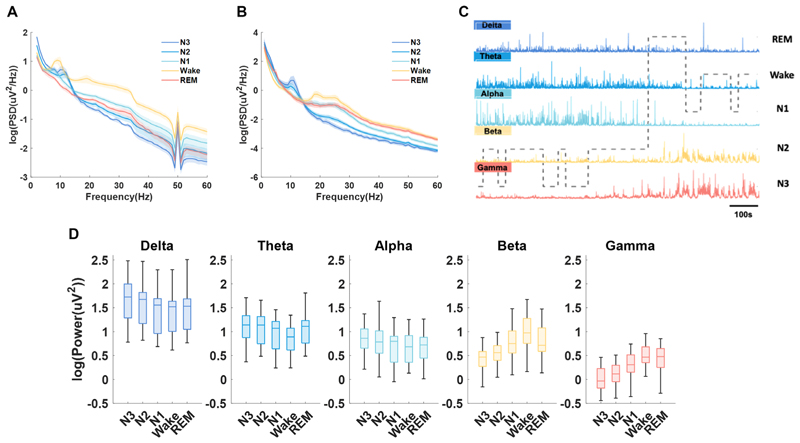
Sleep-stage-dependent characteristics of subthalamic nucleus (STN) local field potential (LFP) and electroencephalogram (EEG). (A) Averaged power spectrum densities (PSDs) (mean±SEM) of different sleep stages from C3 and C4 channels of EEG. (B) Averaged PSDs (mean±SEM) of different sleep stages from LFP. The PSD results were averaged for all hemispheres. (C) Illustration of how delta, theta, alpha, beta and gamma oscillations in STN LFPs changes with time and across different sleep stages in one exemplar patient. (D) Box-and-whisker plots depicting changes in the power of delta, theta, alpha, beta and gamma frequencies during different sleep stages. The box edges represented the first quartile to the third quartile, with a vertical line drawing through the box at the median.

Through simultaneous PSG and STN LFP recording, characteristics of STN LFPs in different sleep stages were investigated. Average PSD of the central EEG and STN LFPs for different sleep stages is shown in figure 3a and b, respectively. In STN LFP, frequency band of delta, theta and alpha showed elevated power with increasing sleep depth from Wake to NREM stages and was highest during deep sleep stages (N2 and N3). In contrast, activities in beta and gamma bands presented an opposite pattern, which were highest during Wake compared with other sleep stages (figure 3d). In REM sleep, the power of beta and gamma frequency bands was elevated compared with N2 and N3 stage, while no significant differences were shown between N1 and REM in the power of any of the frequency bands. In addition, no statistical significance was observed in the frequency band power of REM and Wake comparison, except for theta band, which was slightly higher in REM than in Wake stage. The results of statistical analysis were listed in online supplemental table 3.

### Increased subthalamic beta and low gamma band activities contributed to sleep fragmentation

**Figure 4:**
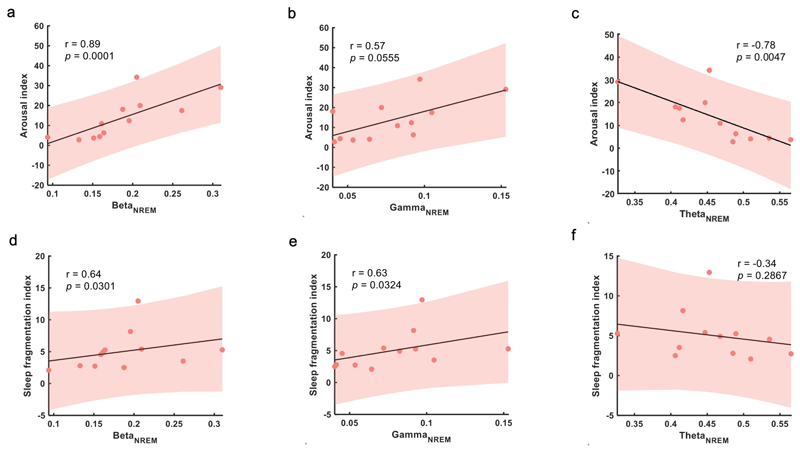
Correlation between sleep fragmentation severity and subthalamic nucleus (STN) local field potential (LFP) oscillations. (A–C) Correlations between arousal index and average power of beta, gamma and theta oscillations during NREM sleep. (D, E) Correlations between sleep fragmentation index and average power of beta, gamma and theta oscillations during NREM sleep. In (A–F), each dot represents data from one participant (the LFP features were averaged across the two hemispheres for each participant); the grey solid line and red shading indicate the linear fittings and 95% CIs were shown.

According to previous research, disrupted sleep in MPTP-induced non-human primate models of Parkinsonism was partially attributed to pathological neural activities, especially elevated beta oscillations, in BG.^7^ We next verify whether the phenomenon still exists in human STN. We used Arl and SFI to quantify the severity of sleep fragmentation and disturbance. The detailed definition and calculation were listed in online supplemental methods. In order to remove the confounding factor that the average power of different frequency bands was modulated by sleep stage, we considered the average power of different frequency bands for REM and NREM separately. The analyses showed that ArI and SFI correlated positively with average power of beta oscillation in NREM sleep (ArI-Beta_NREM_: r=0.9, p=0.0001; SFI-Beta_NREM_: r=0.6, p=0.0301; figure 4a and d). The same trend was also found in low gamma oscillations (SFI-Gamma_NREM_: r=0.6, p=0.0324; figure 4e). On the contrary, negative association was found between average theta oscillation power in NREM sleep and ArI (ArI-Theta_NREM_: r=−0.8, p=0.0047; figure 4c). However, in REM sleep, only average power of low gamma oscillations was found to be positively correlated with SFI (SFI-Gamma_REM_: r=0.6, p=0.0428; online supplemental figure 2e).

**Figure 5:**
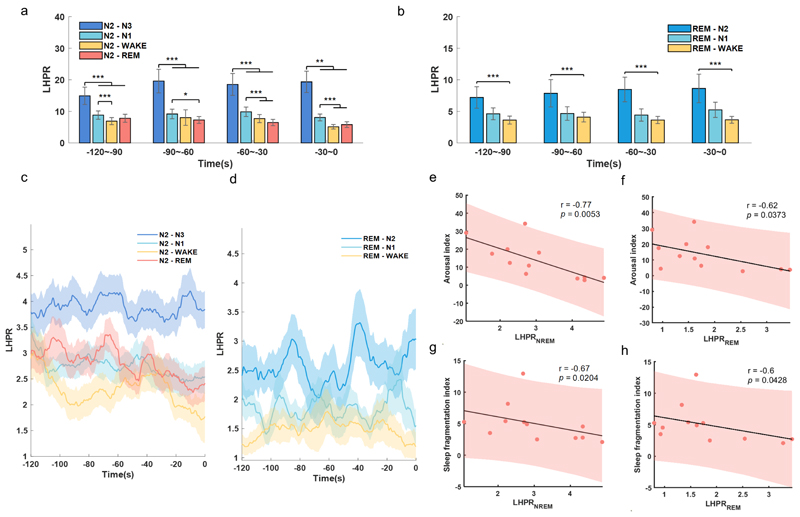
Analysis of low-to-high power ratio (LHPR) during N2 and REM transition process. (A) Comparison of LHPR preceding N2–N3, N2–N1, N2– Wake and N2–REM transitions. (B) Comparison of LHPR preceding REM–N2, REM–N1 and REM–Wake transitions. In both panels A and panel B, the bar graphs illustrated the mean±SEM of LHPR for all hemispheres in non-overlapping 30 s epochs, from 120 s to 0 s before completion of the transitions. **P<0.01; ***p<0.001; (C, D) LHPR changes with time before different transitions from N2 and REM, respectively. In (C) and (D), time 0 indicates the change of sleep stage label, the solid lines and shades represent the mean±SEM across hemispheres. (E, F) Correlations between sleep fragmentation index/arousal index and the average LHPR of NREM sleep. (G, H) Correlations between sleep fragmentation index/arousal index and average LHPR of REM sleep. In (E–H), each dot represents data from one participant (LFP features were averaged across the two hemispheres for each participant); the grey solid line and red shading indicate the linear fittings and 95% CIs were shown.

We further investigated how beta and low gamma band activities participated in sleep fragmentation. As sleep is a dynamic process, the occurrence of a particular sleep stage will be influenced by the transition processes of the previous sleep stage. Thus, not only the typical reduction in N3 sleep and an increase in wakefulness and N1 sleep, but the transition processes, such as N2 to N1, N2 to N3 and so on, highlight their significance in elucidating the process of sleep fragmentation. We focused on the neurophysiological correlates of the transition processes during N2 and REM stages. In particular, we focused on the 120 s of data during N2 and REM just before switching to other sleep stages. We categorised the sleep transition process from N2 to N3 (N2–N3), N1 (N2–N1), Wake (N2–Wake), N2 (N2–REM), as well as REM to N2 (REM–N2), N1 (REM–N1), Wake (REM–Wake). The sample size of events in each transition condition is listed in online supplemental table 3). Considering the inverse relationship of low-frequency and high-frequency band power with sleep fragmentation, low-to-high power ratio (LHPR) of STN LFPs, which is the ratio of the total power of low-frequency oscillations (theta) divided by the total power of high-frequency oscillations (beta and gamma), were analysed and compared for each type of transition. In both N2 and REM transition analysis, statistical significance was shown in each of the non-overlapping 30s epochs before the transition completed. The LHPRs were higher preceding the transition into deeper sleep stages, as during N2–N3 and REM–N2 process, compared with the similar time window preceding the transition to lighter sleep stages (figure 5a and b). To explore the changes with more refined time resolution, LHPR at each time step (with 10-s moving window for each calculation and 0.1-s step length) preceding the sleep stage transition was plotted. As shown in figure 5c, the LHPR gradually reduced with time before both N2–REM, N2–N1 and N2–Wake transitions, but kept high in N2–N3 transitions (figure 5c). In REM transition, the LHPR remained higher in REM–N2 transition comparing with REM–N1 and REM–Wake transitions (figure 5d). Overall, the average LHPR in both NREM and REM sleep negatively correlated with ArI (LHPR_NREM_: r=−0.8, p=0.0053; LHPR_REM_: r=−0.6, p=0.0373) and SFI (LHPR_NREM_: r=−0.7, p=0.0204; LHPR_REM_: r=−0.6, p=0.0428) (figure 5e–h), suggesting that the lower the LHPR in STN LFP during sleep, the higher the sleep fragmentation in PD.

### Beta burst preceded transition to sleep stages with more cortical activity and interfered with sleep spindles

**Figure 6:**
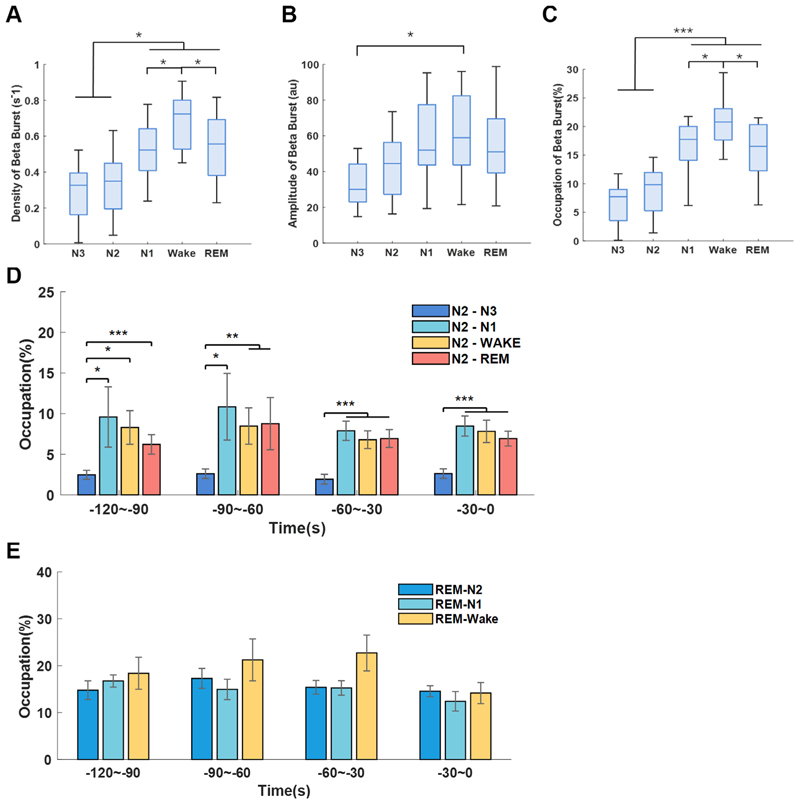
Long beta burst of subthalamic nucleus (STN) local field potential (LFP) during sleep. (A–C) The density (number of events per second), duration and occupations of long beta bursts (>0.25 s) during different sleep stages. The box edges represent the first quartile to the third quartile, with a vertical line drawn through the box showing the median of all hemispheres. *P<0.05; ***p<0.001. (D) Comparison of long beta bursts occupations between N2–N3, N2–N1, N2–Wake and N2–REM transitions. (e) Comparison of long beta bursts occupations between REM–N2, REM–N1, REM–Wake transitions. In (D) and (E), the bar graphs illustrate the mean±SEM of long beta bursts occupations for all hemispheres in non-overlapping 30 s epochs, from 120 s to 0 s before completion of the transitions. No statistical significance was shown.

Long beta burst (>0.25 s, briefly referred as beta burst) has been found to be a pathological biomarker of bradykinesia and rigidity in PD. However, whether it also participated in sleep disturbance in PD remains unknown. Here, we found beta burst can still be detected during NREM, even though the density and occupation of beta bursts in N1, Wake and REM stages were significantly higher than N2 and N3 stages (p<0.05) (figure 6a–c). Beta bursts during different transitions in N2 and REM were then analysed. During the last 120 s preceding the completion of N2 transition, the occupations of beta bursts were higher before transiting to stages with more cortical activity (N2–Wake, N2–N1 and N2–REM) than preceding the transition to deeper sleep stage (N2–N3 transitions) (p<0.01; figure 6d). No differences in beta bursts were observed for different REM transitions (figure 6e).

**Figure 7:**
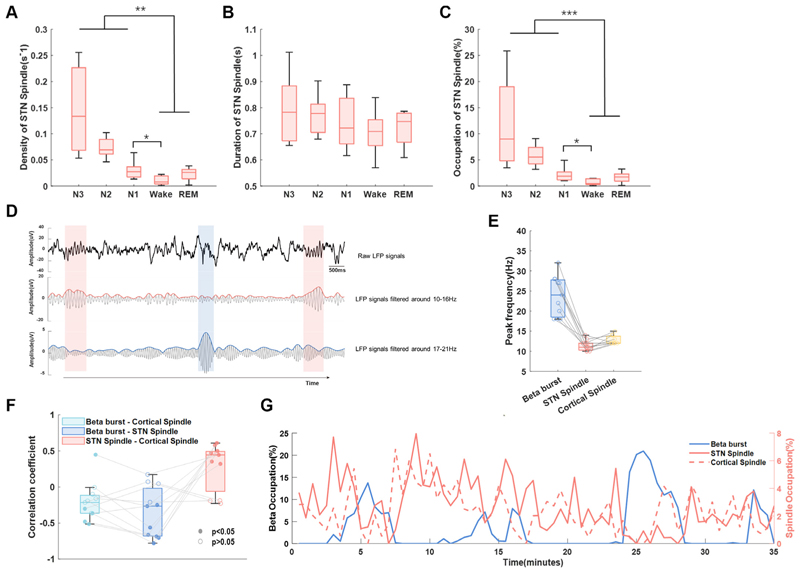
Interaction between long beta burst and sleep spindles. (A–C) The density, duration and occupations of sleep spindles of subthalamic nucleus (STN) local field potential (LFP) in different sleep stages. The box edges represent the first quartile to the third quartile, with a vertical line drawn through the box showing the median of all hemispheres. **P<0.01; ***p<0.001. (D) Example of detected sleep spindles and long beta bursts of STN LFP during NREM sleep in one hemisphere. The detected beta burst and spindles were marked by blue and red shadow, respectively. These three subplots showed the raw LFP signal (top), the 10–16 Hz bandpass filtered signal and its envelope (middle), and the 17–21 Hz bandpass filtered signal and its envelope in sequence (bottom). (E) The peak frequency of beta and sleep spindles in both STN and cortex was distinguishable from each other. (F) The Spearman correlation coefficients between the occupation of STN beta burst and cortical spindle, STN beta bursts and STN spindle, as well as STN spindles and cortical spindles, respectively, for hemispheres in which both prominent beta and spindles were detected. The filled dots represent the coefficients with a p value less than 0.05 while the unfilled dots represent coefficients with a p value greater than 0.05. (G) A typical example of the occupation of long beta bursts, STN spindles and cortical spindles changes over time during NREM sleep from a single subject. The occupations of spindles and beta bursts were averaged over each 120 s window and concatenated during NREM sleep.

As we observed both beta bursts and sleep spindles in NREM sleep, we explored how they interacted with each other. We compared the central frequency of beta burst and spindles in both cortex and STN, and found a clear distinction in the peak frequency ranges among beta bursts (23.8 Hz) and spindles in both STN (11.5 Hz) and cortex (C3-M1 channel, 12.7 Hz) (figure 7e). In addition, average occupations (in percentage of time) of cortical and subthalamic spindles as well as beta bursts were calculated over each 120 s window. LMM identified negative correlations between the occupation of beta bursts and spindles in both STN (β=−0.24, p<0.001) and cortex (β=−0.15, p<0.001) (figure 7f and g).

## Discussion

In this study, we characterised neurophysiological features of STN LFPs underlying sleep fragmentation and abnormality. These results offer new insights into the mechanisms of sleep fragmentations in PD and provide guidance on further interventions.

### Features in STN LFPs underlying sleep fragmentation and insomnia

How neural activities in the subcortical structures contribute to sleep disorders remains largely unknown. Here, we found that the average power of both beta and low gamma oscillations in STN LFPs during NREM sleep was positively correlated with the severity of sleep fragmentation. This is consistent with the behavioural correlation between the motor impairment (UPDRS III scores) and N3 sleep duration and continuity, suggesting that sleep fragmentation in PD can be related to motor impairments and its neural biomarker. These results were also partially consistent with the previous observations from MPTP-primate models that increased beta oscillations in the STN LFP were associated with sleep fragmentation and decreased NREM sleep.^7^ Besides, as theta oscillations in STN LFP have been reported to be positively associated with cognitive functions^22 23^ interruption in theta oscillation could be another mechanism underlying sleep disturbance.

On the other hand, only average power of low gamma oscillations in REM sleep was found to be positively correlated with SFI. Since both STN activities in different frequency bands were modulated by sleep stages, we additionally calculated the correlation of SFI and beta, theta and low gamma oscillations in NREM and REM separately (online supplemental figure 4–5). This generated similar findings: beta and low gamma oscillations in NREM correlated positively with sleep fragmentations severity while only low gamma oscillations in REM demonstrated the same trend (online supplemental figure a,b,e). This can be attributed to a few reasons which need further investigation. First, REM sleep is not a uniform state and can be divided into phasic and tonic substages.^24^ Phasic REM, with enhanced widespread thalamocortical synchronisation activities, demonstrated higher ArI threshold with decreased gamma oscillations than tonic REM.^25 26^ Here, we raised the following hypothesis which need further exploration: the rhythms of phasic and tonic REM can be interrupted in Parkinsonian patients with sleep fragmentation, leading to more tonic REM with lower arousal threshold and higher gamma oscillations. Second, previous research found that in chronic sleep deprivation states, enhanced gamma oscillations were modulated by theta activities in compensation for the losses of REM sleep-related synaptic potentiation.^17^ Thus, the elevated gamma oscillations in STN LFP may also be a compensatory mechanism of sleep disturbance in Parkinsonian patients.

In summary, our results indicated that the alterations of multiple frequency bands in STN LFPs are involved in sleep disturbance in Parkinsonian patients and combining the LHPR in STN LFPs during sleep may serve as a better biomarker for sleep fragmentation.

Further research would be required to dissect how the oscillatory features in the STN LFPs identified in this study interfered with normal sleep: either through serving as a direct neuropathological biomarker of the neural circuits controlling sleep and wakefulness, or by aggravating nocturnal motor symptoms, cognitive impairment and so on.

### Sleep spindles distinguishable from beta bursts in STN LFPs during sleep

As a hallmark of NREM sleep, sleep spindles have an overlap in frequency band with beta oscillations in normal NREM sleep according to previous researches.^27 28^ A previous study conducted during the perioperative period demonstrated the presence of spindle activity in STN LFPs that synchronised with EEG spindle activity.^29^ What is more, recent in vivo studies with non-human primates also demonstrated field potential spindles in BG, which increased during sleep after a learning task especially in the striatum.^10^ Here, in our analysis, we revealed that beta bursts and spindles were distinguishable in terms of frequency and exhibited a negative correlation in their occupation over time for the first time. We confirmed this finding from the following two aspects. First, the peak frequency of beta bursts and sleep spindles in STN was dissociable from each other, with beta bursts showing higher frequency and shorter durations compared with sleep spindles. Second, STN sleep spindles were similar with cortex sleep spindles in peak frequency as well as sleep-stage-dependent distribution density. Moreover, we contend that it is unlikely to identify STN spindles originating from neighbouring pathological oscillation bands, since the spindle activities were absent during wake. However, due to the limited synchronisation accuracy of PSG and LFP recording systems, further researches should aim to provide more details about the directionality of information flows between cortical and STN spindles. As sleep spindles have a close relationship with learning and memory,^30^ previous studies have found that decreased spindle amplitude and density measured from cortex using EEG were associated with dementia in PD.^31 32^ Furthermore, the negative correlation between the occurrence of sleep spindles and beta burst in STN LFPs during NREM sleep indicated that besides contributing to sleep fragmentation, beta bursts may also have detrimental effects on spindle-related physiological functions such memory consolidation during sleep.

Further research would be required to investigate whether STN spindles, together with above-mentioned theta oscillations, could be potential indicators for memory consolidation during sleep and more detailed pathophysiological mechanism of beta oscillations during this process.

### Implications on closed-loop DBS

Closed-loop DBS modulations adjust the stimulation parameters according to the detected biomarkers dynamically,^33^ among which beta power triggered closed-loop DBS in PD have been increasingly studied these days and have been found achieving similar or even better therapeutic effect on motor symptoms comparing with traditional open-loop DBS modulation.^34–36^ However, the effect of beta-triggered closed-loop DBS on sleep is unknown and the closed-loop DBS modulation strategy for sleep is less explored. Previous studies have conducted sleep stage classifications based on STN LFPs and provided possibilities towards more precise neuromodulation around sleep–wake cycles.^9 11^ A recent study on the diurnal fluctuations in beta amplitude suggests that the threshold needs to be adjusted in beta-triggered closed-loop DBS to prevent suboptimal stimulation at night.^12^ Here, in this study, we showed that with the same threshold defined during awake, beta bursts could still be detected during NREM when patients are in off-DBS and off-medication state. Moreover, occurrence of the beta bursts in N2 sleep preceded the transition to stages with more cortical activity (Wake, N1 and REM). These results suggest that, beta power triggered closed-loop DBS, using the same threshold defined during awake, may also reduce sleep fragmentation and improve sleep quality. Another implication of our results on the design of closed-loop DBS is that we may need to differentiate pathological beta oscillation from the sleep spindles. Here, we chose beta within the 15–30 Hz range to maximise the differentiation from the spindle frequency band. The more commonly used beta range of 13–30 Hz was also analysed to confirm the robustness of our conclusions (online supplemental figure 6). Further studies would be required to investigate the effect of high-frequency STN DBS on sleep spindle and related functions, such as overnight learning and memory consolidation.

### Limitations And Caveats

There are several limitations in our study. First, as all data were acquired in off-medication and off-DBS status, whether these trends were stable under different medication and DBS stimulation states remained unknown. Second, as a small-sized clinical study, only 12 patients were included. Interestingly, in this small-sized clinical study, we observed significant correlation between the sleep efficiency of this single recorded night and cognitive function, as measured by the score of MMSE (figure 2c); as well as a negative correlation between N3 percentage and motor impairment as measured by UPDRS III scores (figure 2e). This is consistent with numerous studies suggesting that sleep quality was correlated with cognitive function^37–39^ and DBS can improve both motor symptoms and sleep quality.^40 41^ However, further studies including more subjects and employing more validated sleep scales and sleep diaries before and post DBS, with and without dopaminergic medication, would be required to further verify the association between sleep fragmentation and motor and cognitive impairment in PD, and to better understand the underlying neural features and the impact of DBS or dopaminergic medication on sleep fragmentation in PD.

## Conclusion

In conclusion, our data revealed features of STN LFP underlying sleep fragmentation in PD. These results deepen our understanding of the mechanism of sleep fragmentations in PD and offer new insight on how to improve closed-loop DBS in sleep modulation.

## Supplementary Material

Supplementary Materials

## Figures and Tables

**Table 1:** Demographics of participants

No.	Gender	Age	Disease duration	H-Y^*^	Estimated presurgery LEDDs (mg per day)	UPDRS III (off medication) †				MMSE§				
						Preoperation	DBS off‡	DBS on		Preoperation	1 month	Tremor (DBS off)	Record contacts	Treatment contacts
1	M	53	13	3	189	34	41	11.5		25	28	0/0	2+4-/7+8-** † †	C+3-/C+5-
2	M	67	8	3	800	20.5	16.5	2.5		27	26	0/0	2+4-_+_/6+8-	C+3-/C+7-
3	M	51	20	4	469	20	51	15.5		27	27	2/2	2+4-/5+7-** † †	C+3-/C+6-
4	M	65	6	3	649	21	26	11.5		28		1/3	2+4-/5+7-	C+3-/7+8-
5	F	60	7	3	800	28.5	20.5	9		28	27	0/0	2+4-_+_/6+8-** ††	C+4-/C+7-
6	M	46	7	4	201	46.5	36	17.5		29		0/0	1+3-+/5+7-** ††	C+2-/C+6-
7	F	61	8	4	620	29	33	15		22	24	2/0	2+4-_+_/5+7-	C+3-/C+8-
8^¶^	M	59	6	10	200	8				28		0/0		
9	F	51	6	1.5	500	7.5	5.5	2		28	29	0/5	1+3-^**^† †/7+8-	4+3-/C+8-
10	F	47	12	5	810	38	37	18		28	29	2/2	2+4-^**^ † †/7+8-	C+3-/C+5-
11	M	56	15	4	300	50.5	40.5	18		26	28	2/2	1+3-^*^† †/5+7-^**^ † †	C+2-/C+6-
12	M	61	8	4	326	41	41.5	24		30	29	1/1	1+3-^**^† †/5+7-^**^† †	C+2-/C+6-
13	F	51	8	4	443	53.5	51.5	12.5			26	0/0	2+4-^**^† †/5+7-_+_	C+3-/ C+6-

* H-Y: Hoehn and Yahr Scale

† UPDRS III (off medication): the score was average of the results assessed by two movement disorders specialists according to the Unified Parkinson’s Disease Rating Scale III (UPDRS III), and the difference of scores from the two specialists was less than 10%.

‡ DBS off: the evaluations were conducted before initial programming.

§ MMSE: Minimum Mental State Examination.

¶ 8: Subject 8 was excluded from the subthalamic nucleus (STN) local field potentials (LFPs) analysis due to abnormal impedance of the contacts (>100 kΩ), but was included in the analysis of sleep parameters.

** Periodic oscillatory activities in spindle frequency band were identified.

†† Periodic oscillatory activities in beta frequency band were identified. LEDD, levodopa equivalent daily dose.

## Data Availability

Data are available upon reasonable request. Requests for access to data should be addressed to the corresponding author.

## What is Already known on this Topic

⇒ Beta oscillation, which is a biomarker for rigidity and bradykinesia during awake, significantly reduced during NREM sleep compared with REM or awake. Researches from MPTP (1-methyl-4-phenyl-1,2,3,6-tetrahydropyridine) non-human primate model suggested increased beta oscillation in basal ganglia contributed to insomnia in Parkinson’s disease (PD); however, evidence in human patients is still lacking.

## What this study adds

⇒ Beta and low gamma band activities in subthalamic nucleus (STN) local field potentials (LFPs) during sleep recorded from human patients with PD correlated with severity of sleep impairment. The low–high power ratio can serve as a biomarker for sleep fragmentation. Besides, pathological beta bursts and physiological sleep spindles can be detected in STN LFP during NREM sleep and distinguishable from each other.

## How this study might affect research, practice or policy

⇒ These findings enhance our understanding of the electrophysiological mechanisms that underlie sleep fragmentation in PD. The study also has implications on the design of closed-loop DBS: we may need to take multiple frequency band activities into consideration and differentiate pathological beta oscillation from the sleep spindles.
